# Comparison of dwarf bamboos (*Indocalamus* sp.) leaf parameters to determine relationship between spatial density of plants and total leaf area per plant

**DOI:** 10.1002/ece3.1728

**Published:** 2015-09-30

**Authors:** Pei‐Jian Shi, Qiang Xu, Hardev S. Sandhu, Johan Gielis, Yu‐Long Ding, Hua‐Rong Li, Xiao‐Bo Dong

**Affiliations:** ^1^ Collaborative Innovation Center of Sustainable Forestry in Southern China of Jiangsu ProvinceBamboo Research InstituteNanjing Forestry University159 Longpan RoadXuanwu DistrictNanjing210037China; ^2^ Institute of Food and Agricultural SciencesEverglades Research and Education CenterUniversity of FloridaBelle GladeFlorida; ^3^ Departement Bio‐ingenieurswetenschappenUniversity of AntwerpGroenenborgerlaan 171B‐2020AntwerpBelgium

**Keywords:** Data fitting, density, Gielis equation, leaf shape, log‐linear, self‐thinning rule

## Abstract

The relationship between spatial density and size of plants is an important topic in plant ecology. The self‐thinning rule suggests a −3/2 power between average biomass and density or a −1/2 power between stand yield and density. However, the self‐thinning rule based on total leaf area per plant and density of plants has been neglected presumably because of the lack of a method that can accurately estimate the total leaf area per plant. We aimed to find the relationship between spatial density of plants and total leaf area per plant. We also attempted to provide a novel model for accurately describing the leaf shape of bamboos. We proposed a simplified Gielis equation with only two parameters to describe the leaf shape of bamboos one model parameter represented the overall ratio of leaf width to leaf length. Using this method, we compared some leaf parameters (leaf shape, number of leaves per plant, ratio of total leaf weight to aboveground weight per plant, and total leaf area per plant) of four bamboo species of genus *Indocalamus* Nakai (*I. pedalis* (Keng) P.C. Keng, *I. pumilus* Q.H. Dai and C.F. Keng, *I. barbatus* McClure, and *I. victorialis* P.C. Keng). We also explored the possible correlation between spatial density and total leaf area per plant using log‐linear regression. We found that the simplified Gielis equation fit the leaf shape of four bamboo species very well. Although all these four species belonged to the same genus, there were still significant differences in leaf shape. Significant differences also existed in leaf area per plant, ratio of leaf weight to aboveground weight per plant, and leaf length. In addition, we found that the total leaf area per plant decreased with increased spatial density. Therefore, we directly demonstrated the self‐thinning rule to improve light interception.

## Introduction

Bamboos of *Indocalamus*, or dwarf bamboos are common wild plants in the rural areas of southern China. Bai et al. (2011) report that *Indocalamus longiauritus* can dominate forest understory and function as an ecological filter. The genus can also provide habitats for birds and lizards. For example, Chinese bamboo partridge (*Bambusicola thoracica*) is often observed to act around these bamboos (Liu et al. 2012), and its Chinese name exactly reflects the apparent relationship between this species and the bamboos. The planting of these dwarf bamboos is now extended to parks, campus, and other public places in cities of southern China. Dwarf bamboos are highly resistant to cold (Tian et al. 2006), hence some species of *Indocalamus* have been introduced to the 40°N parks, and they can safely overwinter without any special care in China (Wang and Chen 2012). Kobayashi (2015) reports that the northern distribution limits of some species of genus *Sasa* can reach 50°N in Japan. Considering the increasing air dust pollution in northern China, it is valuable to introduce dwarf bamboos to northern cities. In fact, trees and grasses give only (very low vegetative covering) rate for many northern cities in winter because many trees are deciduous and many grasses in lawns are not evergreen. Because of the lack of water and poor resistance to cold, many grasses in lawns in northern cities consume a large number of water and labor resources but exhibit low efficiency in providing fresh oxygen and reducing the dusts in the air from vehicles and construction industry. In addition, the common lawn grasses are easy to be invaded by other species, so they also require additional labor for weeding. Relative to common lawns, bamboo lawns are not easy to be invaded. Many dwarf bamboos rapidly occupy the sites of common grasses and replace them.

Leaves in bamboo are of diverse nature. The growing stem is protected by culm sheath with a restricted blade (cataphylls), whereas the upper leaves have a fully developed blade. These leaves are the uppermost leaves of the plant, which develop after culms elongate to full height and in *Indocalamus* species can be up to 40 cm long and 5–8 cm wide. Like in (almost) all woody and herbaceous the leaves are connected to the leaf sheath through a petiole, often referred to as pseudopetiole (McClure 1966), a constriction zone, which allows the positioning of the leaf blade through torsion. These structures, found in the whole subfamily of the bamboos, have been demonstrated to be an advantageous solution for adapting to forest environments.

Leaves are photosynthetic organs of plants, therefore, the shape and size of leaves has marked effects on the success of plants (Tsukaya 2006). The leaf shape of different crops has long been attracting the attention of researchers (Sanderson et al. 1981; Bos et al. 2000; Dornbusch et al. 2011). These studies mainly provide two‐dimensional planar leaf‐shape models for comparative analysis of leaf shape among different crops or among different developmental stages of the same crop in the growing season. Some studies address the dynamic change of three‐dimensional leaf shape (Zhu et al. 2009; Miao et al. 2013; Aksoy et al. 2015). However, the three‐dimensional modeling for leaf shape is still based on the planar leaf‐shape models. For example, Aksoy et al. (2015) use an ellipse to describe the leaf shape of tobacco plants, whereas Dornbusch et al. (2011) develop a multiparametric model to describe the axial‐symmetric convex pentagon of grass leaf shape. The early models are well applicable to the leaf shape of the studied plants. For bamboos, given that bamboo leaf blades are very similar throughout the whole subfamily, we need a more suitable model describing the leaf shapes. Gielis (2003) puts forward a general formula (that we will refer to as “Gielis equation” hereafter) based on the superellipse equation, which can be used to describe many shapes of biological organs, such as flowers, tree rings, starfish shells, and cobwebs. Only a few studies have paid enough attention to the function of this formula on describing the leaf shapes of plants (Wang 2007). In the present study, we proposed a simplified Gielis equation to fit the leaf‐shape data of these four species, and compared the difference in an important parameter in this simplified Gielis equation that could reflect the overall ratio of leaf width to leaf length.

Total leaf area per plant is important for exploring the efficiency of photosynthesis. However, for many trees, the number of leaves is too large to accurately estimate the total leaf area per tree. For common grasses, the part of leaf surface especially around the leaf bottom is not flat, so it is rather difficult to accurately calculate their total leaf area. However, the bamboos of *Indocalamus* sp. have <50 leaves per plant, with clear leaf profile and its surface is basically flat from bottom to tip. It provides us with an opportunity to compare the difference in total leaf area among different species and to link such a difference to other biological indicators, e.g., the aboveground fresh weight per plant, biomass per unit, the spatial density per unit that might be affected by leaves’ photosynthesis.

The self‐thinning rule is an important ecological rule in describing the relationship between body size in plants and spatial density. It is first proposed to depict the relationship between stem density and quadratic mean diameter at breast height in a pure, even‐aged stand (Reineke 1933). Then, it is widely used to describe the relationship between mean biomass and density of the population (Yoda et al. 1963; White and Harper 1970; Lonsdale 1990). There is a debate on the −3/2 power between mean biomass and plant density (Lonsdale 1990; Han and Fang 2008). In fact, the self‐thinning phenomena also occur in plant organs e.g., stem, branch, and leaf yield (Xue and Hagihara 2008). Westoby (1977) finds that −3/2 self‐thinning is driven by leaf area rather than weight. He uses *Helianthus annuus* to demonstrate there is an obviously −3/2 power between mean leaf area per plant and density (plants per m^2^). His study shows that mean leaf area is a better indicator for exhibiting the self‐thinning rule. However, of the above studies related to self‐thinning rule none except Westoby (1977) consider the mean leaf area per plant. Although some studies use leaf area, the results are not very convincing because of the lack of robust methods for accurately estimating the total leaf area per plant. As many studies use trees or herbaceous plants with irregular leaf shape, it is rather difficult to estimate the total leaf area per plant.

In this study, we aimed to compare the leaf parameters (overall ratio of leaf width to leaf length, total leaf area per plant, ratio of leaf weight to aboveground weight per plant) among four species of *Indocalamus* to find the relationship between these parameters and spatial density (i.e., plants per m^2^). In addition, we tested whether the relationship between leaf fresh weight and leaf area was approximately linear. If the approximate linear relationship holds, we check whether the slopes of leaf area plotted against leaf fresh weight among four close relative species of the same genus have significant difference. Meanwhile, we can calculate the total leaf area per plant by using the total leaf weight per plant if the approximate linear relationship between leaf area and leaf weight holds. Then, we can further examine whether self‐thinning phenomenon between total leaf area per plant and spatial density (namely plants per unit area) exists or not.

## Materials and Methods

### Experimental site

Four species of genus *Indocalamus* Nakai (species‐1: *I. pedalis* (Keng) P. C. Keng; species‐2: *I. pumilus* Q. H. Dai and C. F. Keng; species‐3: *I. barbatus* McClure; species‐4: *I. victorialis* P. C. Keng) (Table 1) were collected along the Verdant Bamboo Road in Nanjing Forestry University (32.08°N, 118.82°E). Nanjing belongs to the subtropical areas. Based on the climate data from 1951 to 2012 (downloaded from the website of China Meteorological Data Sharing Service System [http://cdc.nmic.cn]), the mean annual precipitation was 1058 mm (±237.5 mm standard error), the mean annual temperature was 15.6°C (±0.7°C), the mean minimum annual temperature was −8.6°C, the mean maximum annual temperature was 37.4°C, the mean relative humidity was 75.7%, and the mean annual sunshine duration was 2038 h (±190 h). In general, 5°C is usually defined to be a threshold temperature for calculating the accumulative heat sum for plant growth and development (e.g., Holdridge 1947; Diekmann 1996; Fang and Lechowicz 2006). Thus, we calculated the mean annual accumulative heat sum of Nanjing from 1951 to 2012 using the daily air temperature data. It is 4100 degree‐days (±199 degree‐days) per year in Nanjing.

**Table 1:** Agronomic and morphological characteristics of four bamboo species of genus *Indocalamus*

Species	Leaf length (cm)	PN	PW	Total leaf area (cm^2^)	Leaf number	Total leaf weight (g)	Weight (g)	Height (cm)
1	18.4 ± 5.6	<0.01	0.391	549.0 ± 317.6	11.0 ± 6.2	6.53 ± 3.90	18.1 ± 9.80	71.6 ± 23.2
2	14.0 ± 4.1	<0.01	0.149	658.3 ± 363.0	23.4 ± 13.2	7.80 ± 4.42	19.7 ± 13.2	68.1 ± 35.4
3	14.7 ± 4.2	<0.01	0.182	694.2 ± 403.2	21.4 ± 13.6	6.83 ± 4.00	18.7 ± 8.7	88.8 ± 26.6
4	17.0 ± 4.3	<0.01	0.262	861.4 ± 526.4	14.2 ± 9.8	10.6 ± 6.42	29.2 ± 14.5	76.5 ± 23.5

Here, leaf length was estimated based on individual species without distinguishing different plants of the same species; PN is the *P*‐value that tests the normal distribution of data on leaf length, whereas PW is the *P*‐value that tests the Weibull distribution of data on leaf length. Other characteristics were measured based on individual plant. Total leaf area per plant was estimated according to (1) the regression coefficients of leaf area to leaf fresh weight and (2) the leaf number per plant (see eqs (3) and (5)).

### Experimental design and data acquisition

We chose 90 plants of every species randomly from their habitats in early July of 2014 when the shape and size of leaves of these bamboos were basically invariable. The aboveground height and fresh weight were measured for each plant. All leaves per plant were clipped for weighing the total fresh weight per plant by using an electronic scale with precision 0.01 g (JM‐A3002; Chaozeheng Equipment Company Limited, Zhuji, Zhejiang, China). Number of leaves per plant and the length of each leaf were also recorded. We also randomly chose more than 100 leaves from different plants of every bamboo species (that are different from the above selected 90 plants), and measured the length and fresh weight of each leaf. Furthermore, we scanned every leaf shape using a photo scanner (HP Scanjet 4850; Hewlett‐Packard Company, Palo Alto, California) and obtained its bmp (Bitmap) image. Furthermore, we extracted the leaf‐shape data from the bmp image using a MATLAB program proposed by Shi et al. (2015). For every leaf, the number of boundary point ranged 1500–5000, which depended on the resolution of bmp image. In general, 500 data points were enough to obtain a clear profile of a leaf.

To obtain the data of spatial density (plants in m^2^), we used a 1 × 1 ‐m plot for *I. pedalis*,* I. pumilus*,* I. barbatus*, and a 2 × 1 ‐m plot for *I. victorialis* because of the sparsest spatial distribution. Every species had five replicates (i.e., five plots). The mean of densities from five plots was used to represent the density for every species.

### Data analysis

In order to compare the overall ratios of leaf width to leaf length among four species, we proposed a simplified Gielis equation based on the original (Gielis 2003):

(1) $$r=\frac{l}{{\left(\left|\cos\frac{\varphi }{4}\right|+\left|\sin\frac{\varphi }{4}\right|\right)}^{1/n}}$$

Here, *r* = *x*/cos *φ* = *y*/sin *φ*, where *x* and *y* represent the Cartesian coordinates of the profile data of a scanned leaf, and *r* and *φ* represent their polar coordinates. Apparently, *r* = *l* when *φ *= 0, *n* is a parameter that can determine the overall ratio of leaf width to leaf length that will be referred to as the leaf‐shape parameter hereafter. The leaf length (*L*) is the sum of *r*, when *φ *= 0 and *r*, when *φ = π*:

(2) $$L=\left(1+2^{-\frac{1}{2n}}\right)\cdot l$$

As a result of this simplication there are only two parameters in this special case of the Gielis equation. Although parameter “*n*” can affect the calculation of leaf length (*L*) in theory, the determinant for *L* is parameter “*l*”. When *n* ranges from 0 to 0.15, the ratio of *l* to *L* is higher than 0.90 (Fig. S1), “*n”* is the determinant for the overall ratio of leaf width to leaf length. Then our interest was to compare the leaf‐shape parameters among four species. The Tukey's Honest Significant Difference (HSD) test was used to compare the estimates of parameter “*n*” on the leaf samples from different bamboo species (>100 leaves for each species).

As these four species came from the same genus, their relationship was expected be close. We checked whether there were significant differences in slope among four regression straight lines of leaf area to fresh weight using the covariance analysis (Faraway 2005). Although some studies show that there is an allometric relationship (i.e., the log‐linear relationship) between leaf area and leaf dry weight ($A=\alpha W_{D}^{\beta }$, where *A* represents area of a leaf, *W*_*D*_ dry weight of a leaf, *α* a constant, and *β* a power), the estimates of power for many plants were approximately 1 (e.g., Hagihara et al. 1993; Khan et al. 2005; Milla and Reich 2007). In this case, we could assume an approximate linear relationship between leaf area and leaf dry weight. For plants, there is an obvious proportional relationship between their dry weight (*W*_*D*_) and fresh weight (*W*), i.e., *W *= *ζW*_*D*_, where *ζ* is a constant (Shi et al. 2013b). Consequently, there would be also an approximate linear relationship between leaf area and leaf fresh weight. We carried out a linear fitting to leaf area versus leaf fresh weight for each species:

(3) A=a+bW

Here, *a* and *b* are constants. To show whether this linear approximation is reasonable, we will also carry out a linear fitting to the natural logarithm of leaf area versus the natural logarithm of leaf fresh weight:

(4) ln(A)=c+dln(W)

Here, *c* and *d* are constants. If the linear relationship between leaf area and leaf fresh weight holds, we can calculate the total leaf area per plant (*A*_total_) from the total leaf fresh weight per plant (*W*_total_):

(5) $$A_{\mathrm{total}}=ka+bW_{\mathrm{total}}$$

Here, *k* is the number of leaves per plant. $A_{\mathrm{total}}=\sum_{i\;=1}^{k}A_{i}$ and $W_{\mathrm{total}}=\sum_{i\;=1}^{k}W_{i}$. Then we used the Tukey's HSD test to check whether there was any significant difference in total leaf area per plant (also leaf number per plant) among these four species. Leaf area was scanned and calculated using the polygon method in the special package of “splancs” in the statistical software R (R Development Core Team 2015). We also used the integrated equation (1) to calculate leaf area, and then compared this predicted area with the scanned leaf area, to test the model validity in describing the leaf shape. The calculating principle was similar to that in Shi et al. (2015).

Leaf length was also a concern since it is related to leaf area and leaf weight. We examined whether the data of leaf length in each species followed a normal distribution or a Weibull distribution using the Shapiro–Wilk test and the Kolmogorov–Smirnov test, respectively (Bolker 2007; Xue and Chen 2007).

All analyses were performed by the statistical software R (version 3.1.3; R Development Core Team 2015).

## Results

### Model validation and comparison of leaf‐shape parameter

**Figure 1:**
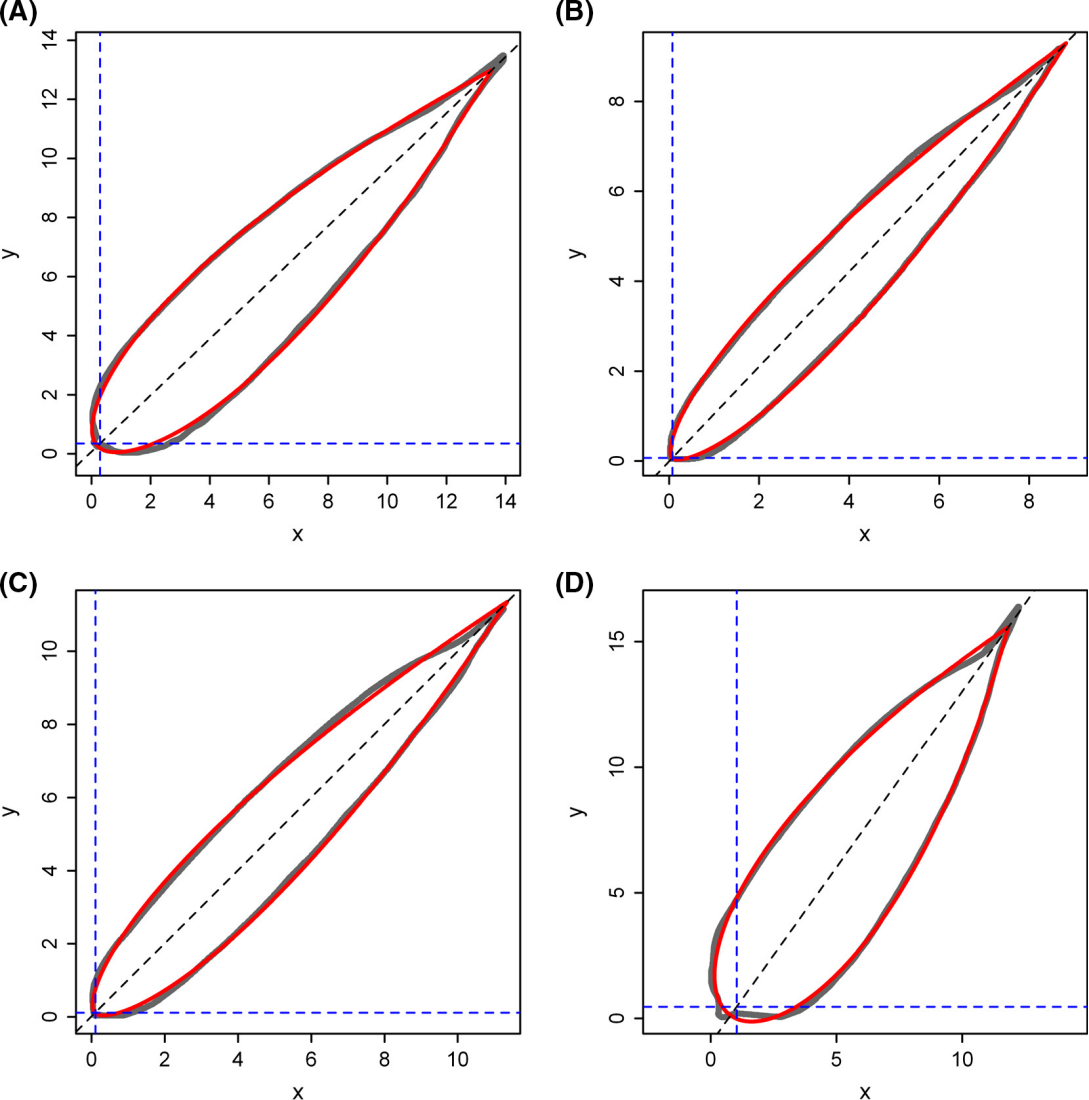
Comparison between the observed and predicted profile of a leaf. Panels (A–D) represent species 1–4, respectively. The gray solid line represents the observed profile, and the red line represents the predicted profile of a leaf using the simplified Gielis equation. Unit: cm.

**Figure 2:**
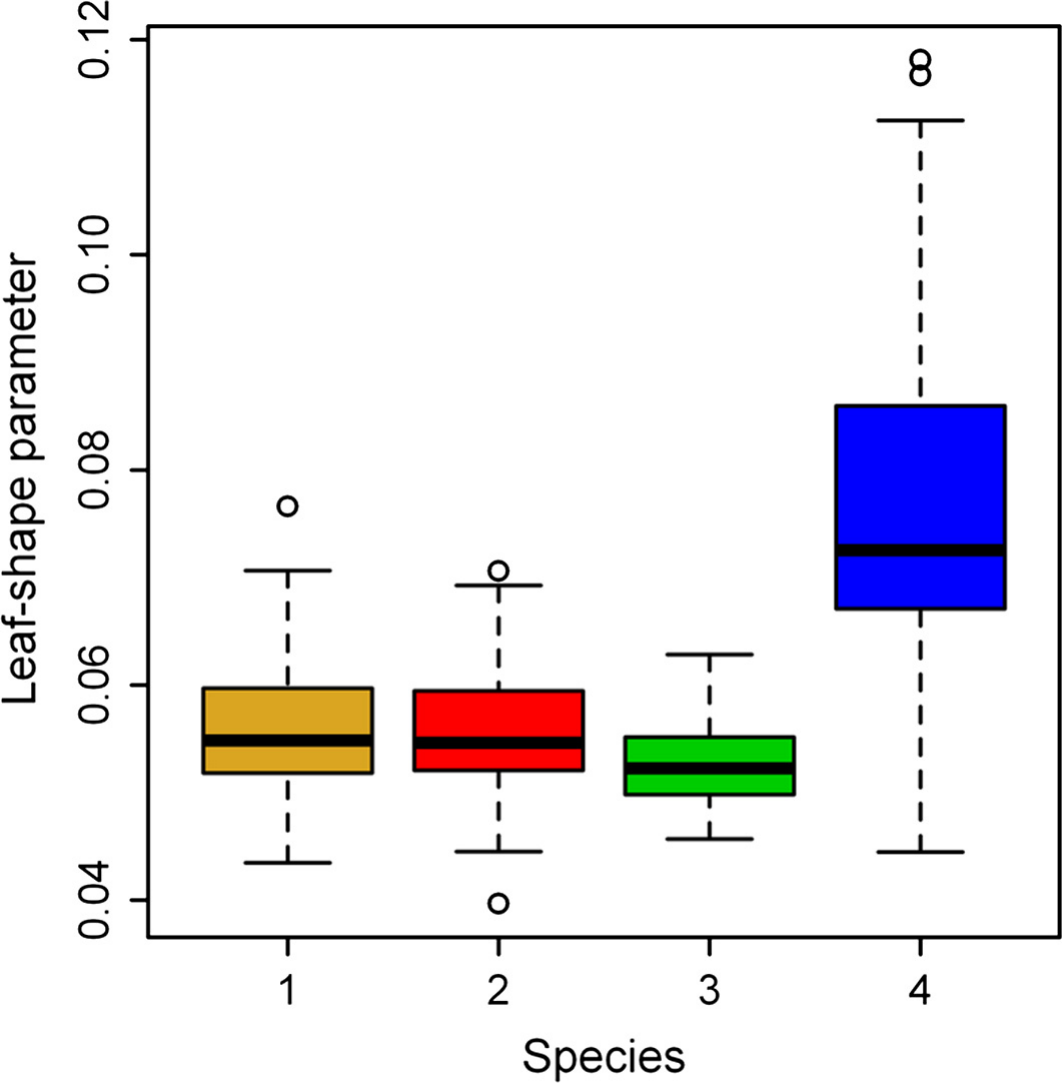
Comparison of leaf‐shape parameter “*n”* among four bamboo species.

Figure 1 exhibited the comparison between observed profile and predicted profile of one sample from each bamboo species. The simplified Gielis equation fits the profile data of all bamboo leaf samples very well (based on the goodness of fit shown in Table S1). Figure 2 showed the comparison of leaf‐shape parameter “*n*” among these four species. There were no significant differences in leaf‐shape parameter among species 1–3, but species 4 (i.e., *I. victorialis*) was significantly different from others. Species 4 has broader leaves than other species. Overall, the estimates of leaf‐shape parameters for four species lied in a small range of 0.03–0.12.

### Comparison of total leaf weight, total leaf area and ratio of total leaf weight to aboveground weight per plant among four species

**Figure 3:**
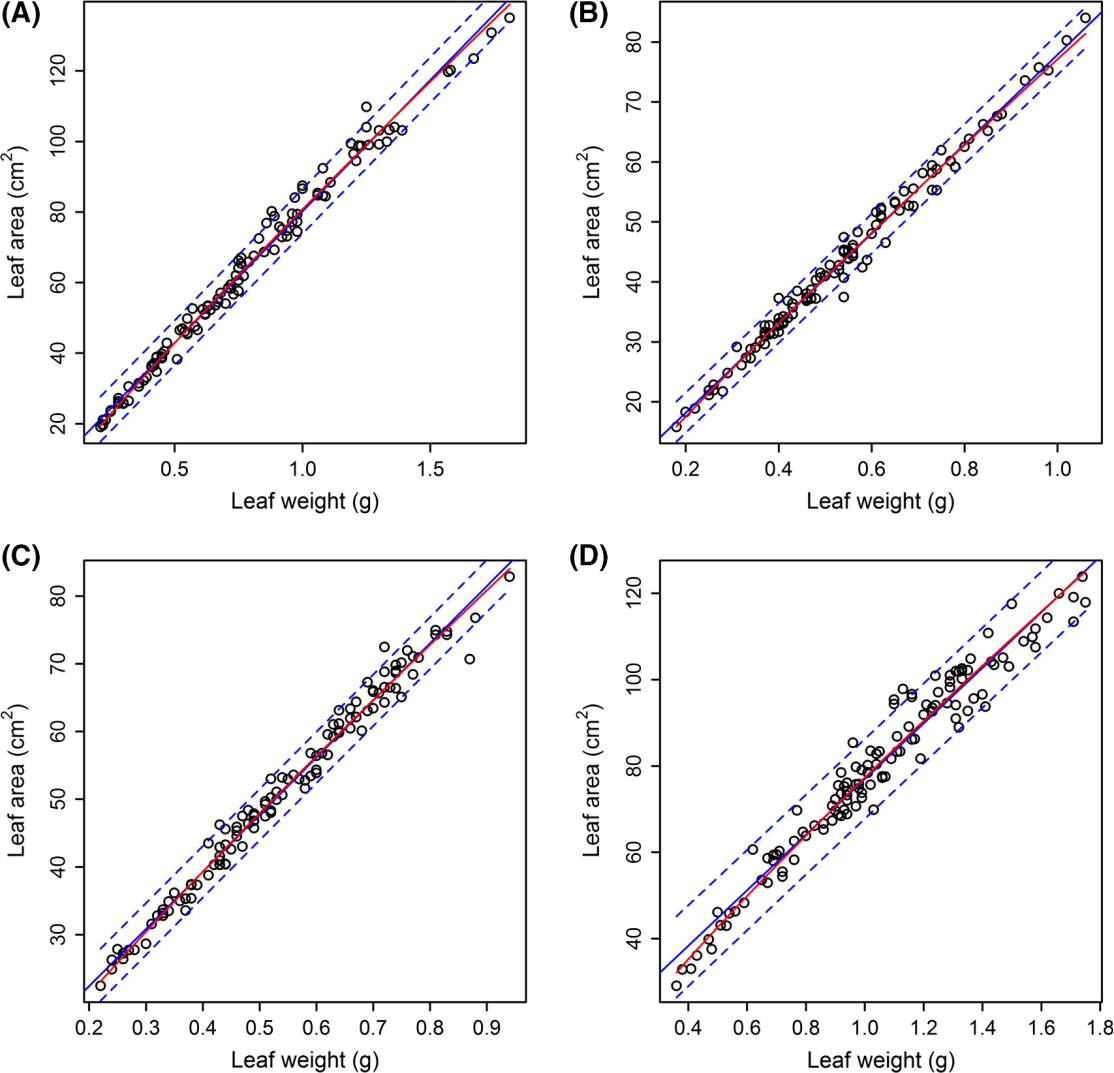
Linear and log‐linear relationships between leaf area and leaf fresh weight. Panels (A–D) represent the fitted results of species 1–4, respectively. The small open circles represent the observed values; the blue straight line represents the regression line of *A = a + b W*; and the two blue dashed lines represent the corresponding 95% confidence intervals; the red straight line represents the regression line of ln(*A*) = *c *+ * d* ln(*W*), where the data of leaf area were expressed to be e^c^ *W* ^d^.

**Figure 4:**
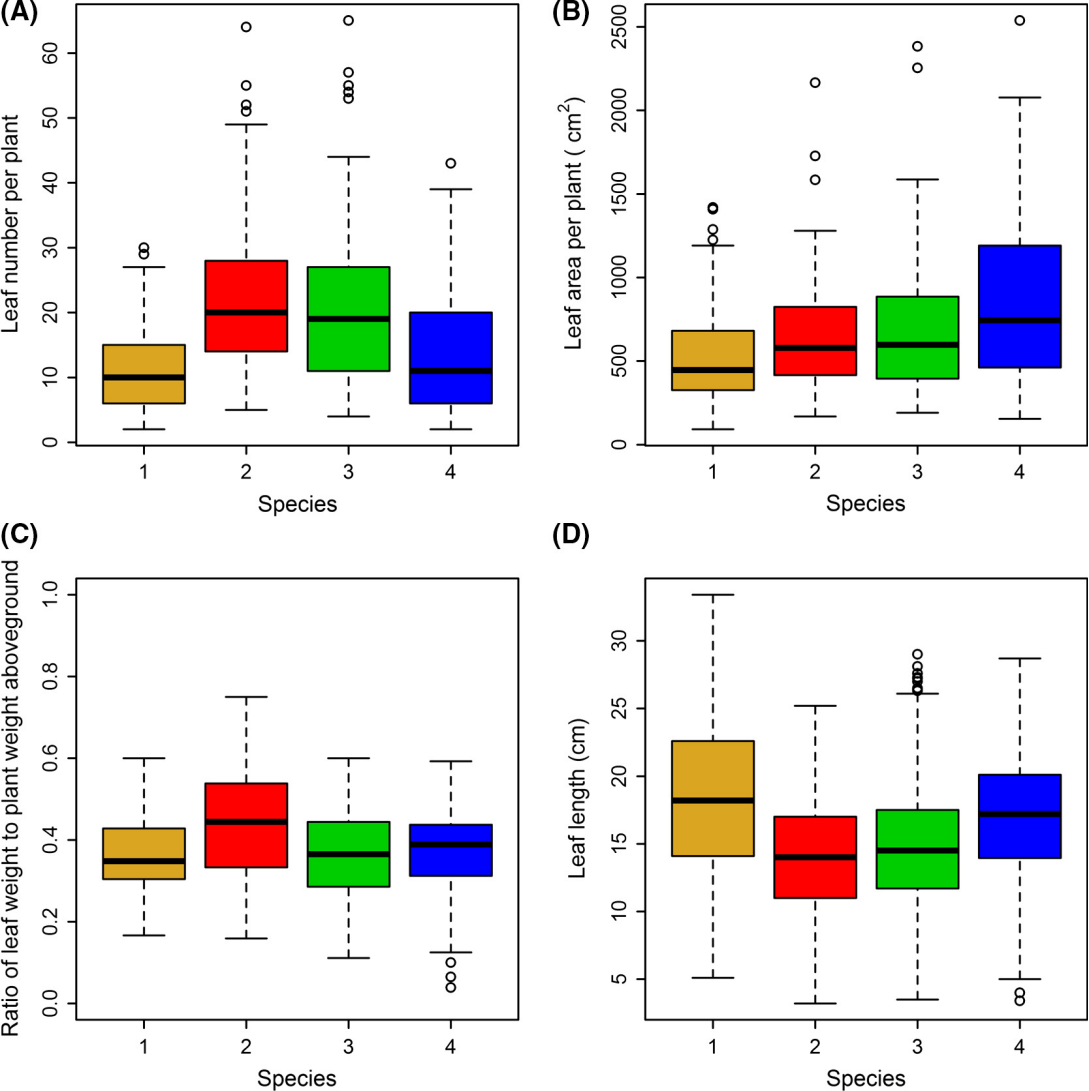
Comparison of leaf number (A), total leaf area (B), ratio of total leaf weight to the total aboveground weight per plant (C), and leaf length (D) among four species.

Figure 3 showed that there was a strong linear relationship between leaf area and leaf fresh weight for every species (see blue straight lines). It was feasible to use the linear relationship to replace the log‐linear relationship, because these two functional relationships produced similar results, which can be examined by observing whether the blue straight line approximates to the red curve. The regression coefficients and corresponding standard errors were listed in Table 2. The linear relationship held for these two basic biological indicators of leaf area and leaf fresh weight. Although the estimates of slope for four species were slightly smaller than 1 from the allometric relationship between leaf area and leaf fresh weight (also see Table 2), the coefficients of determinations for four species by using the linear relationship were very approximate to those by using the log‐linear relationship. Thus, it was feasible to use a linear equation to depict the relationship between leaf area and leaf fresh weight. However, the covariance analysis showed that there were significant differences in slope among these four regression straight lines (Table 3). Because of the existence of strong linear relationship between leaf area and leaf fresh weight, it was feasible to use eqs (3) and (5) further to estimate the total leaf area per plant based on the leaf number per plant and the total leaf fresh weight per plant. Table 1 exhibited these predictions on total leaf area per plant for all four species. Using Tukey's HSD test, we found that the number of leaves in species 2 and 3 were not significantly different from each other, but higher than those of species 1 and 4 (Fig. 4A). There were no significant differences in leaf number between species 1 and 4. However, for total leaf area per plant, only species 4 was significantly different from all others (Fig. 4B). Using Tukey's HSD test, we found that the ratio of total leaf weight to aboveground plant weight of species 2 (with a mean of 44.3%) was significantly different from other three species (with mean ranging from 36 to 37%; Fig. 4C). Overall, the total leaf weight actually occupied a large proportion (>1/3rd) of aboveground weight of the plants in genus *Indocalamus*.

**Table 2:** The linear and log‐linear relationship between leaf area and leaf fresh weight

Species	Sample size	*A = a* +* b W*			ln(*A*)* *=* c *+* d* ln(*W*)		
		Estimate of *a*	Estimate of *b*	*R* ^2^	Estimate of *c*	Estimate of *d*	*R* ^2^
1	112	5.66 ± 0.68	74.50 ± 0.80	0.99	4.3913 ± 0.0052	0.9131 ± 0.0078	0.99
2	108	3.24 ± 0.48	74.68 ± 0.83	0.99	4.3455 ± 0.0080	0.9210 ± 0.0104	0.99
3	113	5.47 ± 0.61	84.48 ± 1.07	0.98	4.4862 ± 0.0073	0.8900 ± 0.0099	0.99
4	121	12.59 ± 1.44	64.39 ± 1.31	0.95	4.3521 ± 0.0055	0.8555 ± 0.0178	0.96

**Table 3:** Covariance analysis of four regression lines of leaf area plotted against leaf fresh weight

Item	Estimate	*t* value	*P‐*value	*F*_(4,449)_	*R* ^2^
Intercept	7.7053 ± 0.5676	13.575	<0.001	5316	0.9793
Leaf fresh weight	71.8137 ± 0.6008	119.531	<0.001		
Species 2	−2.9170 ± 0.4984	−5.852	<0.001		
Species 3	4.6794 ± 0.4921	9.509	<0.001		
Species 4	−2.9420 ± 0.4990	−5.896	<0.001		

We did not test the difference in weight per leaf or area per leaf among these four species. However, to test the validity of the simplified Gielis equation, we compared the calculated leaf area using the polygon method with that using the integral of the simplified Gielis equation. We found that the predicted leaf areas using these two methods basically coincided (Fig. S2).

### Comparison of leaf length and the distributional type

**Figure 5:**
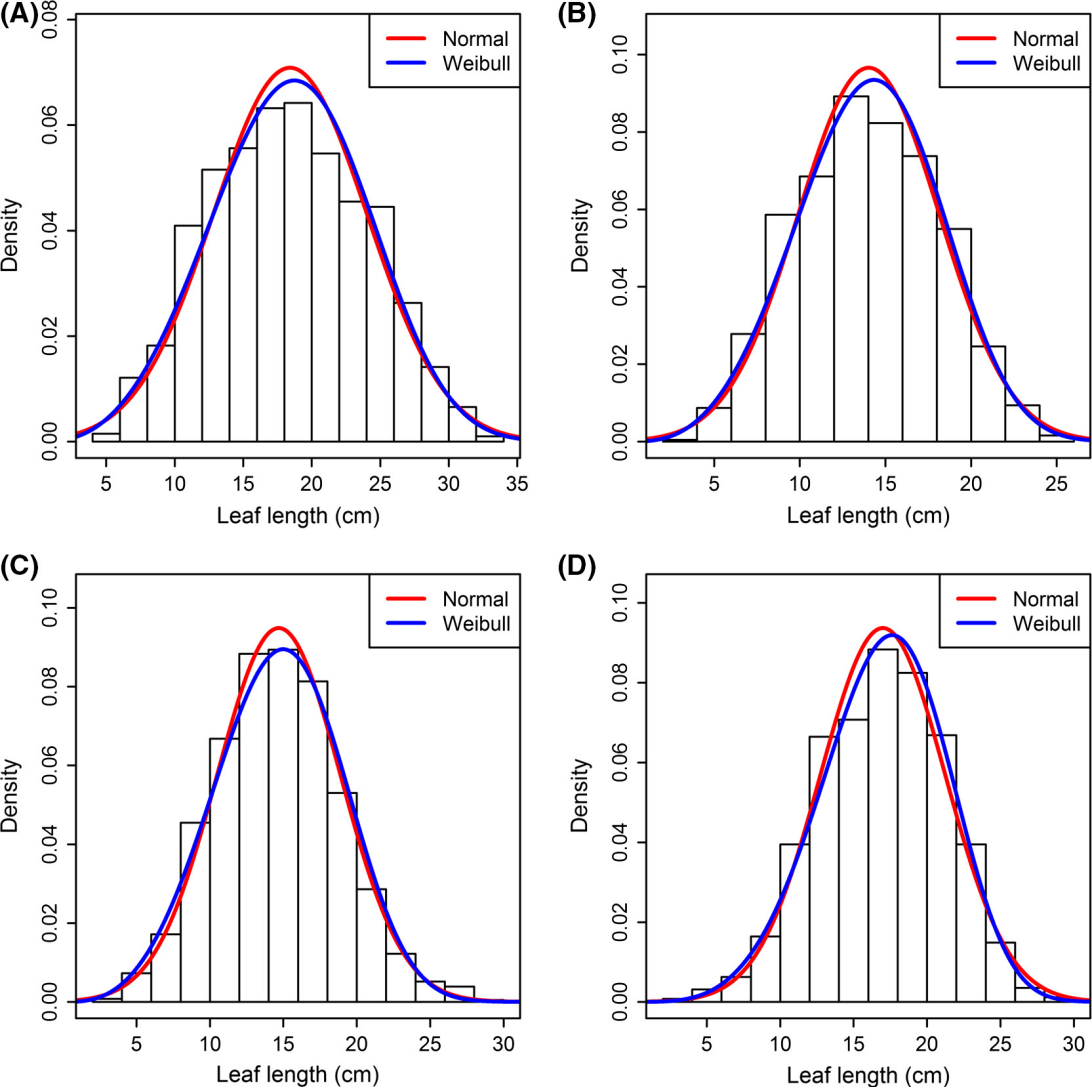
Comparison between the normal distribution and the Weibull distribution in describing the leaf length. Panels (A–D) represent species 1–4, respectively.

Using Tukey's HSD test, we found that there were significant differences in leaf length among these four species (species 1 > species 4 > species 3 > species 2; Fig. 4D). For every species, the normality test's results showed that the leaf length failed to follow a normal distribution (*P *<* *0.05; Table 1). However, they all followed a Weibull distribution (*P *>* *0.05; also Table 1). Figure 5 intuitively exhibited the comparison of fit for two distributional types. Visual inspection suggested that the Weibull distribution matched the observed distribution better than did a normal distribution.

### Comparison of spatial density among four bamboo species

**Figure 6:**
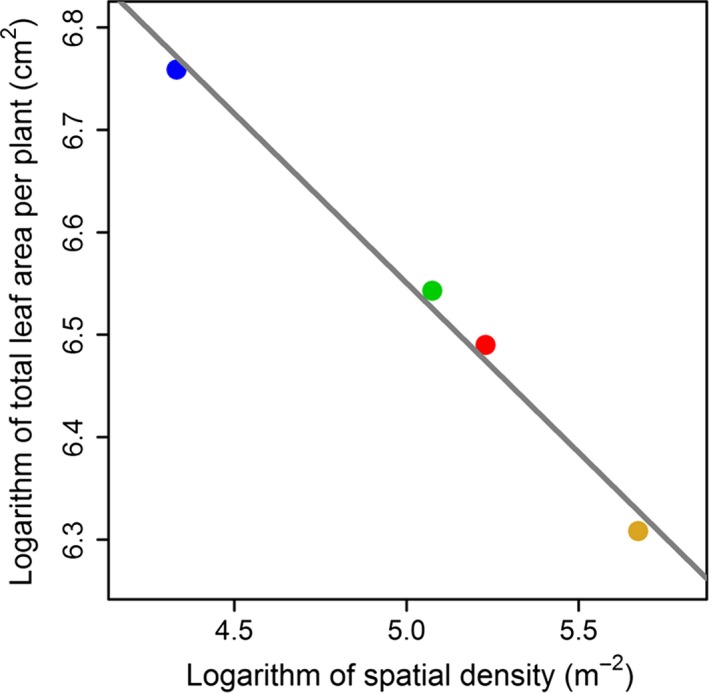
Regression of the logarithm of total leaf area per plant on the logarithm of spatial density of plants. For every species, total leaf area per plant represents the mean of total leaf area per plant among 90 samples, and spatial density represents the mean of observed densities among five plots.

There were on average 291 ± 22 plants of *I. pedalis* per m^2^, 187 ± 16 plants of *I. pumilus* per m^2^, 160 ± 51 plants of *I. barbatus* per m^2^, and 76 ± 19 plants of *I. victorialis* per m^2^. The order of density was: species 1 > species 2 > species 3 > species 4. We performed a log‐linear regression of total leaf area per plant on spatial density, and found a strong log‐linear relationship among them (Fig. 6; estimated slope = −0.331 ± 0.024; *P*‐value < 0.01; *R* ^2^ = 0.990).

## Discussion

The simplified Gielis equation only has two parameters, *n* and *l*, which describe bamboos leaf shape very well. Relative to the previous leaf‐shape models (e.g., Sanderson et al. 1981; Bos et al. 2000; Dornbusch et al. 2011), this model has following advantages: (1) fewer parameters, (2) providing a possibility for comparing these leaves that have similar proportional shapes but different lengths using one parameter “*n*”, (3) more suitable for these leaves whose bottom is convex, flat, and smooth. However, further investigation is required to determine if it is also applicable for the leaf shapes of other grasses. In addition, all the leaf‐shape models almost have not considered the effect of temperature on the leaf shapes in the growing season. The previous studies have demonstrated that temperature can exert pressure on the number of leaves of a plant, the rate of leaf extension, and leaf length (Watts 1971; Peacock 1975; Voorend et al. 2014). In the different developmental stages of rice, the leaf shape has been demonstrated to vary with time (actually thermal time) in the growing season (Zhu et al. 2009). Thus, it is more valuable to explore the leaf‐shape change over the leaf extension season using parameter “*n*” in the simplified Gielis equation. The other parameter “*l*” in this equation approximates the leaf length, and can be regarded as an indicator of size. In fact, temperature can affect the size and growth rate in plants and ectotherms (Shi et al. 2013a). Thus, it was also interesting to explore the effect of temperature on leaf length and leaf area. However, for the plants of *Bambusoideae*, the leaf shape stabilizes after completing the extension. In our investigation dates, all four species had completed the process of extension in spring, so the investigated leaf shapes in summer represented their final shapes. Consequently, the leaf length and leaf area were constant. Leaf fresh weight might be slightly variable during study periods. Therefore, dynamics of leaf weight also deserves further investigation. We did not find that the regression slope of straight lines of leaf area and leaf fresh weight remained constant among four closely related species from the same genus, and the slope variations were also different. Out of four species, “species 4” (*I. victorialis*) was the largest, and its leaf shape exhibited the largest variation in slope. Relative to the leaf length of other three species, “species 4” was not the largest, even the mean was significantly lower than that of species 1 (Fig. 4D).

With regard to the allometric relationship between surface (*S*) and weight for a plant or animal, the power is approximately estimated to be 2/3 (Makarieva et al. 2004 and references therein), that is:

(6) $$S\propto W^{2/3}$$

It comes from a hypothesis that an organic shape is similar to a cube or a sphere of uniform density. It is feasible for a whole plant or animal and some organs similar to a cube or a sphere. However, for a special organ like a leaf, it is very thin, so the power of a leaf between its surface and weight should be 1 ‐ *ε*, where 1/3 ≪ *ε *< 1. Obviously, *S ≈ 2A* for a leaf, and consequently the power between its area and weight is also 1 ‐ *ε*. The previous studies have demonstrated that there is an allometric relationship between leaf area and leaf dry weight (Hagihara et al. 1993; Khan et al. 2005; Milla and Reich 2007) for trees. They also demonstrate that the estimates of power are slightly smaller than 1 (namely the estimate of *d* in eq. (4)). Our study also showed that the estimates of powers for four species of bamboos were smaller than 1. However, the allometric relationship between leaf area and leaf fresh weight cannot be directly used to calculate the total leaf area per plant. We demonstrated that using the linear equation can also reflect the relationship between leaf area and fresh weight. The log‐linear equation is slightly better than the linear equation, but the former can be approximated by the latter. It is easy to explain this approximation by using the first‐order Taylor series expansion of *W* ^*d*^ at the mean of sampled leaf areas ($\overset{¯}{W}$). Equation (4) can be rewritten as:

(7) $$\begin{array}{cc} A\; & =\;e^{c}W^{d}\\ & =e^{c}\left(\frac{{\overset{¯}{W}}^{d}}{0!}+\frac{d{\overset{¯}{W}}^{d-1}}{1!}\left(W-\overset{¯}{W}\right)+\ldots \right)\\ & \approx e^{c}\left(1-d\right){\overset{¯}{W}}^{d}+\left(e^{c}d{\overset{¯}{W}}^{d-1}\right)\cdot W \end{array}$$

Then, we can obtain the approximate values of coefficients in equation (3):

(8) $$\left\{\begin{array}{c} a\approx e^{c}\left(1-d\right){\overset{¯}{W}}^{d}\\ b\approx e^{c}d{\overset{¯}{W}}^{d-1} \end{array}\right.$$

Due to the limited space, we did not discuss it further. If we knew the number of leaves of a plant and the total fresh weight of leaves of a plant, we could directly calculate the total leaf area of the plant by using equation (5). Milla and Reich (2007) have demonstrated that *d *<* *1, so *a *>* *0 (Note: Milla and Reich (2007) use 1/*d* rather than direct *d*, so their conclusion is 1/*d *>* *1). Thus, the intercept *a* in equation (3) cannot be neglected when describing the linear relationship between leaf area and leaf fresh weight.

**Figure 7:**
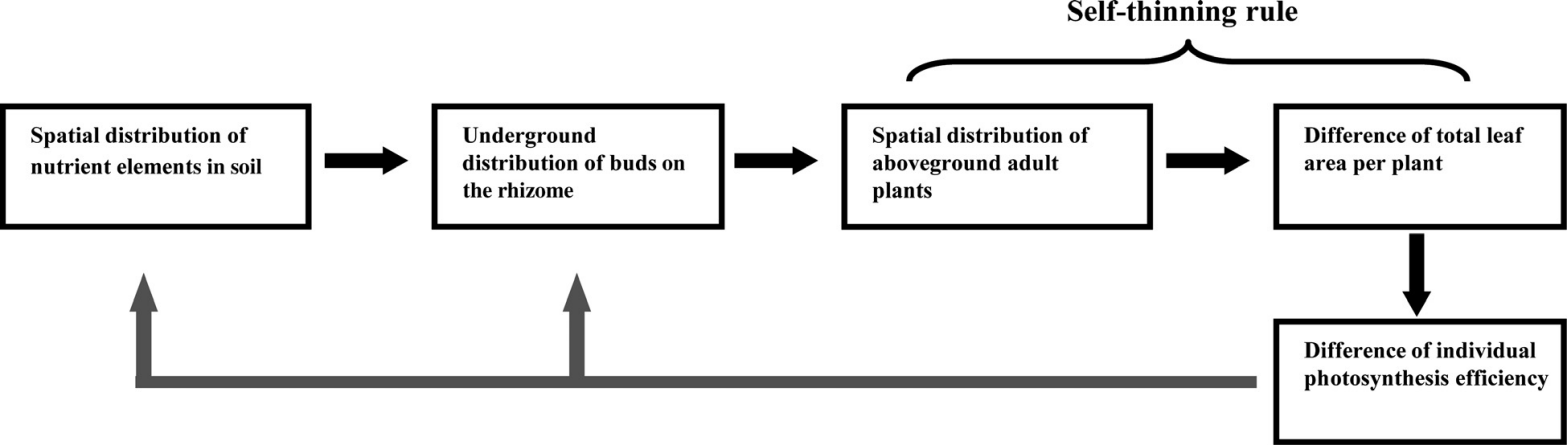
Illustration of the theoretical position for the self‐thinning rule between spatial distribution and total leaf area per plant.

The self‐thinning rule is one general ecological principle in plant population biology (Yoda et al. 1963; White and Harper 1970; Lonsdale 1990). It reveals that −3/2 power rule relates the average plant biomass to the spatial density. It also can be transferred to a −0.5 power rule related to the stand yield and the spatial density (Lonsdale 1990). The theoretical reasons for this phenomenon have been interpreted by previous studies (e.g., White 1981; Li et al. 2000). The current study coincided with the prediction of self‐thinning, because the total leaf area per plant actually had a strong linear relationship with leaf weight. It illustrated that leaf weight per plant also follows the self‐thinning rule. Our study demonstrated that the self‐thinning rule also applied to the total leaf area per plant. It demonstrated the postulation of White and Harper (1970) that there is probably a self‐thinning rule for the plant parts (leaves, roots, etc.). The leaf area relates to the light competition, so the self‐thinning rule occurs (Lonsdale and Watkinson 1982). In fact, the theoretical logic seems to be reasonable if we use the total leaf area per plant to explain the self‐thinning rule between spatial density and average weight. Because of light competition, the total leaf area in a given region should be limited. Then individual plants have to adjust their leaf area for co‐existence. For dwarf bamboos, the case seems to be more complex to us. We here provided an analytical theoretical framework for explaining the status of self‐thinning rule between spatial density and total leaf area per plant (Fig. 7). In general, the herbaceous plants exhibit clustering in space due to the concentration difference of nutrient elements. Nutrient‐rich environment favored species with a high specific leaf area to high leaf weight ratio (Poorter and Remkes 1990). Our study implicated that the clustering might be progressively strengthened by the individual difference in total leaf area per plant, because it might lead to the aggravation of spatial heterogeneity of nutrient gradient in soil due to the difference of individual photosynthesis efficiency. The transport of nutrients in the rizome occurs from their area of high concentration to low concentration (Li 2015). However, the neighboring areas of high nutrient concentration area are usually benefited due to easy access. Thus, the herbaceous plants are prone to cluster according to the spatial distribution of nutrients in soil. The real ecological goal is to explore the underlying process of the interactions among individuals and between species that produce different patterns (Li et al. 2000). We considered that the difference of total leaf area per plant renders the self‐thinning rule more stable in space. In a given region, the total leaf area of all plants should be limited, which further affects the recruitment of the next generation, and it also determines the final average biomass and total biomass of plants (i.e., the stand yield) in a given region. From this sense, our study is very important because it demonstrated the self‐thinning rule between total leaf area per plant and the spatial density. Also, our study showed a linear relationship between them using logarithmically transformed data. We demonstrated that the self‐thinning phenomenon between total leaf area per plant and density also existed among different species that are closely related in taxon. In fact, Liu et al. (2015) have demonstrated that there is a self‐thinning rule between the ground diameter and density among 50 species of bamboos. As all bamboos with different body size belong to the same subfamily, they are actually closely related in taxon and in evolution. The close species share the same or similar ecological niche and have similar environmental requirements, so they look more like “one single species”. It merits further investigation on the self‐thinning rule using close related species that belong to the same taxon unit, e.g., family, subfamily, genus, even subgenus.

## Conflict of Interest

None declared.

## Supporting information


**Figure S1.** Effects of parameter “*n”* on the ratio of parameter “*l”* to leaf length “*L”*. Here, *n* and *l* are parameters in the simplified Gielis equation.
**Figure S2.** Comparison between the “observed” leaf area and the “predicted” leaf area.

**Table S1.** Parametric estimate of leaf shape using the simplified Gielis equation.
