# Supplementary figures and images for: Comparison of dwarf bamboos (Indocalamus sp.) leaf parameters to determine relationship between spatial density of plants and total leaf area per plant
Source: Ecol Evol. 2015 Sep 30;5(20):4578–89. doi: 10.1002/ece3.1728 (PMC4670054; doi:10.1002/ece3.1728)

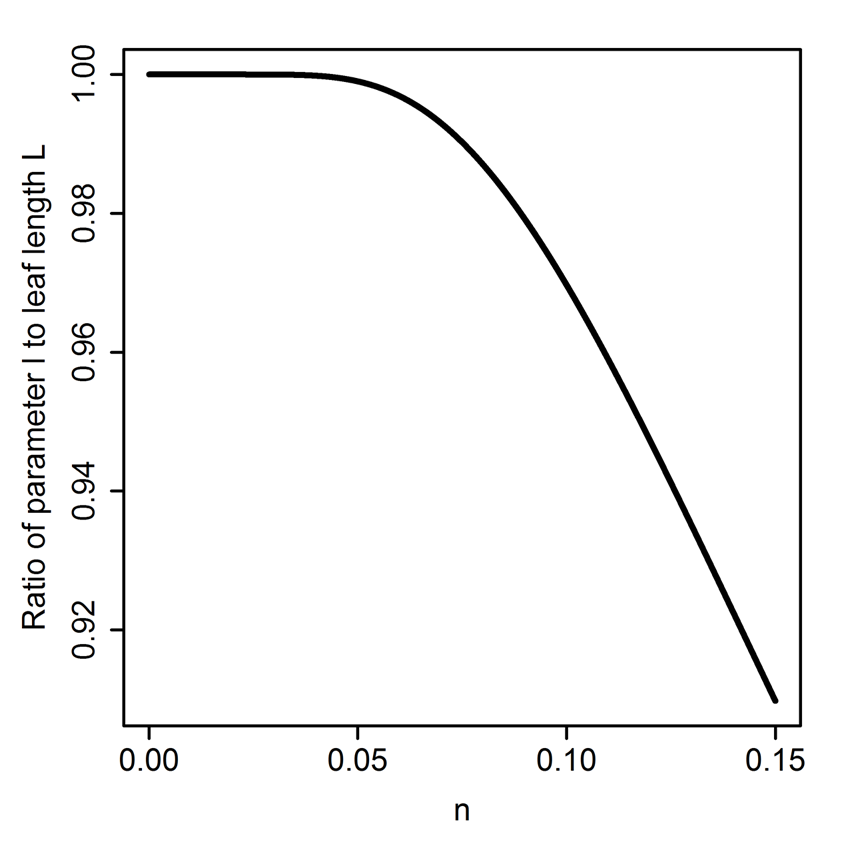


**Figure S1.**


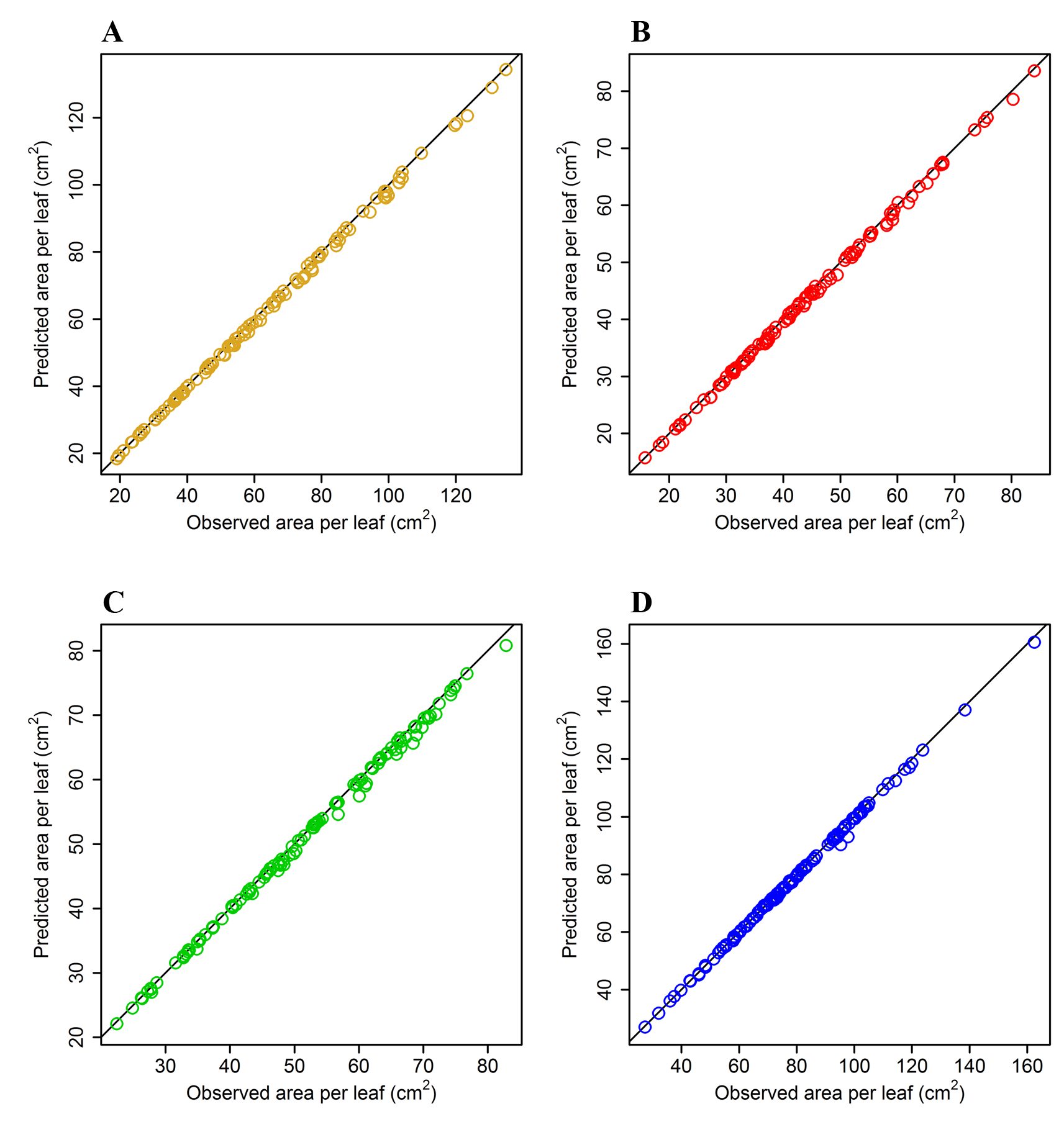


**Figure S2.**

Supplement: Supplementary file 1 — Figure S1. Effects of parameter “n” on the ratio of parameter “l” to leaf length “L”. Here, n and l are parameters in the simplified Gielis equation. Figure S2. Comparison between the “observed” leaf area and the “predicted” leaf area. [file ECE3-5-4578-s001.docx]
